# Quantitative Trait Loci Associated with the Tocochromanol (Vitamin E) Pathway in Barley

**DOI:** 10.1371/journal.pone.0133767

**Published:** 2015-07-24

**Authors:** Ryan C. Graebner, Mitchell Wise, Alfonso Cuesta-Marcos, Matthew Geniza, Tom Blake, Victoria C. Blake, Joshua Butler, Shiaomen Chao, David J. Hole, Rich Horsley, Pankaj Jaiswal, Don Obert, Kevin P. Smith, Steven Ullrich, Patrick M. Hayes

**Affiliations:** 1 Department of Crop and Soil Science, Oregon State University, Corvallis, Oregon, United States of America; 2 Cereal Crops Research, USDA-ARS, Madison, Wisconsin, United States of America; 3 Department of Botany and Plant Pathology, Oregon State University, Corvallis, Oregon, United States of America; 4 Plant Sciences and Plant Pathology Department, Montana State University, Bozeman, Montana, United States of America; 5 Crop Improvement and Genetics Research, USDA-ARS, Albany, California, United States of America; 6 Busch Agricultural Resources, Inc., Fort Collins, Colorado, United States of America; 7 USDA-ARS Cereal Crops Research Unit, Fargo, North Dakota, United States of America; 8 Plants, Soils, and Climate Department, Utah State University, Logan, Utah, United States of America; 9 Department of Plant Sciences, North Dakota State University, Fargo, North Dakota, United States of America; 10 Limagrain Cereal Seeds, Lafayette, Indiana, United States of America; 11 Department of Agronomy and Plant Genetics, University of Minnesota, St. Paul, Minnesota, United States of America; 12 Department of Crop and Soil Science, Washington State University, Pullman, Washington, United States of America; Zhejiang University, CHINA

## Abstract

The Genome-Wide Association Studies approach was used to detect Quantitative Trait Loci associated with tocochromanol concentrations using a panel of 1,466 barley accessions. All major tocochromanol types- α-, β-, δ-, γ-tocopherol and tocotrienol- were assayed. We found 13 single nucleotide polymorphisms associated with the concentration of one or more of these tocochromanol forms in barley, seven of which were within 2 cM of sequences homologous to cloned genes associated with tocochromanol production in barley and/or other plants. These associations confirmed a prior report based on bi-parental QTL mapping. This knowledge will aid future efforts to better understand the role of tocochromanols in barley, with specific reference to abiotic stress resistance. It will also be useful in developing barley varieties with higher tocochromanol concentrations, although at current recommended daily consumption amounts, barley would not be an effective sole source of vitamin E. However, it could be an important contributor in the context of whole grains in a balanced diet.

## Introduction

The tocochromanols—including α-tocopherol (αT), α-tocotrienol (αT3), β-tocopherol (βT), β-tocotrienol (βT3), δ-tocopherol (δT), δ-tocotrienol (δT3), γ-tocopherol (γT) and γ-tocotrienol (γT3) forms—are credited with protecting polyunsaturated fatty acids from lipid peroxidation [1]. Tocopherol and tocotrienol fractions are differentiated by the level of saturation on the polyprenyl chain. Of these eight tocochromanol forms, αT and γT receive the most attention: αT because it is the most concentrated in human plasma, and γT because it is the most abundant in many typical human diets [2]. While all tocochromanol forms have similar anti-oxidant properties and are in some cases referred to, cumulatively, as “vitamin-E”, αT is the only tocochromanol form that meets the Recommended Daily Allowance (RDA) for vitamin-E, so the term “vitamin-E” commonly refers specifically to αT.

Despite the well-established nutritional requirement of tocochromanols for reproductive health and normal neurological development in mammals [3], the precise physiological function of these compounds remains elusive. The scientific literature is replete with laboratory studies on the nutritional benefits of tocochromanols, particularly with respect to cardiovascular disease [4]. Oddly, depending on the specific health risk, human epidemiological studies have been equivocal [5], with some reporting that the impact of αT is positive [4,6], negative [7], or relatively neutral [8]. In one exceptionally large trial, in which 39,876 apparently healthy women were administered either vitamin E or a placebo over an average of 10.1 years, very little evidence was found that vitamin E reduced the risk of either cardiovascular diseases or cancer [9]. However, most of the current literature is based on experiments where supplements, in the form of natural or synthetic αT, were used to test the effects of vitamin E on human health. High doses of αT are known to inhibit absorption of other tocochromanols in humans [10,11], and these effects may be long lasting [12]. More research is needed to fully understand the effects of consuming tocochromanols in a natural form (i.e. in whole grains).

In addition to their possible implications for human health, tocochromanols play an important role in plant stress tolerance. One key function of tocochromanols is to protect lipid membranes in the photosynthetic machinery from a range of oxidative stresses, primarily by deactivating ^1^O_2_ and OH˙ reactive oxygen species [13]. When used to scavenge lipid peroxyl radicals in plants, tocochromanols must be restored by another reducing agent, such as ascorbate (vitamin C) to re-gain functionality. In scavenging ^1^O_2_, the anti-oxidant is irreversibly damaged [13]. The functions of other tocochromanols in plant physiology remain to be elucidated.

To date, there have been two major studies of the genetic controls of tocochromanol synthesis in barley. In one study, the cDNA sequence encoding homogentisate geranylgeranyl transferase (*HGGT*), an enzyme necessary for tocotrienol synthesis, was isolated in barley [14]. In the same study, the barley *HGGT* sequence was used for *Agrobacterium*-mediated transformation of maize, resulting in a six-fold increase of tocotrienols in the seed. However, the gene encoding this cDNA was not assigned a linkage or physical map position. In a more recent study [15], analysis of a bi-parental mapping population resulted in the identification of three Quantitative Trait Loci (QTL) associated with the concentrations of one or more tocochromanol forms in barley, one on chromosome 6H, and two on chromosome 7H. The QTL on chromosome 6H was attributed to *VTE4*, and one of the QTL on chromosome 7H was attributed to either *HGGT* or *VTE2*, based on orthology between rice and barley.

The availability of a comprehensive linkage map and a genome sequence in barley makes it possible to assess which regions in the barley genome are associated with variations in tocochromanol concentration, using a Genome-Wide Association Studies (GWAS) approach. GWAS is now widely used in a range of crop plants and is a powerful tool for rapidly detecting QTL and possibly even specific candidate genes [16,17]. In barley, GWAS has been used to identify QTL related to flowering time [18,19], disease resistance [18], and food quality [18,20].

Our objectives were to a) quantify the concentration of each tocochromanol form in cultivated barley using accessions from eight US spring barley breeding programs, b) identify QTL in the barley genome associated with the concentration of each tocochromanol form and fraction, and c) use identified QTL in conjunction with the barley genome sequence to identify candidate genes.

## Methods

This research was based on 1,534 spring-habit barley accessions from the Barley Coordinated Agriculture Project (Barley CAP), a predecessor to the Triticeae Coordinated Agricultural Project (TCAP; http://www.triticeaecap.org/; verified 26 October 2014). The “Barley CAP I” and “Barley CAP II” germplasm sets consisted of elite breeding lines and varieties from ten breeding programs participating in the Barley CAP: Montana State University (MT), North Dakota State University two-row and six-row (N2 and N6), the USDA-ARS program based at Aberdeen, Idaho (AB), the University of Minnesota (MN), Utah State University (UT), Washington State University (WA), and Busch Agricultural Resources Inc. (BA). The 1,534 spring barley accessions were grown, one time per accession, over a two year period (2006 and 2007) at Bozeman, Montana, USA, as described by Pauli et al. [21]. The grain samples used for this research originated from the irrigated trial in 2006, and the dryland trial in 2007.

Tocochromanols were analyzed and quantitated using a modified saponification method [22]. Approximately 1 g of grain was ground in a Retsch ZM-1 mill (Haan, Germany) and an aliquot (approximately 0.5 g) was weighed and the weight recorded. The freshly-ground sample was then extracted by addition of 0.5 ml 10M KOH, 0.5 ml 95% ethanol, 0.5 ml 0.15M NaCl and 1.25 ml of a 0.5M solution of pyrogallol (in 95% ethanol) and shaken in a water bath at 70°C for 30 min., vortexing every 10 min. The tubes were cooled on ice and an additional 3.75ml of 0.15M NaCl was added. This suspension was extracted twice with hexane/ethyl acetate (9:1 v/v) by vortexing and centrifuging at 1000g for 5 min and transferring the supernatant to a glass test tube. The combined organic phase was reduced to dryness in a Thermo-Savant SPD1010 speed-vac system (Asheville, NC) at 45°C. The dried extract was re-suspended in 1.0 ml hexane and centrifuged to remove particulates prior to analysis by High Performance Liquid Chromatography (HPLC). For HPLC analysis, each sample was analyzed with a Shimadzu LC-5a HPLC (Kyoto, Japan) using a 4.6 × 250 mm, 5 m Adsorbosil silica column (Grace Co., Deerfield IL.) with an isocratic mobile phase at a flow rate of 2.0 ml/min. Samples from the barley CAP I germplasm were separated using a mobile phase of 0.5% isopropanol in hexane. Unfortunately this solvent system did not effectively separate the γT and the βT3 content, thus a different mobile phase consisting of 2% ethylacetate and 2% dioxane in hexane, which did separate these two congeners, was used for the Barley CAP II germplasm. Fluorescence detection was employed using a Shimadzu RF-10A spectrofluorometer with excitation at 295 nm and detection at 330 nm. Peaks were integrated and compared to tocochromanol standards. Tocotrienols were quantitated using the standard curve developed for the corresponding tocopherol [23]. Tocochromanol data for germplasm arrays are available at The Triticeae Toolbox (T3) (http://triticeaetoolbox.org/; verified 13 October 2014) [24].

Barley accessions in the “Barley CAP I” and “Barley CAP II” germplasm arrays were genotyped for 3,072 single nucleotide polymorphism (SNP) markers with two GoldenGate Olionucleotide Pool Assays (OPAs), as described by Close et al. [25] and Szücs et al. [26]. The genotyping was conducted at the USDA-ARS Small Grains Genotyping Center in Fargo, North Dakota. After excluding markers with more than 10% missing data and markers that were cosegregating in this set of germplasm, 2,204 of the 3,072 SNP markers from the two OPAs were used in this analysis. Of the 1,534 accessions genotyped, 68 were excluded from the analysis because they had more than 10% missing genotypic data. Therefore, the GWAS is based on 1,466 barley accessions. SNP data was retrieved from The Triticeae Toolbox (T3) (http://triticeaetoolbox.org/; verified 13 October 2014) (Blake et al. 2012).

Linkage map positions from the barley consensus map [27] were used to identify the position of SNP markers. One SNP marker that was significant in this analysis, 11_20311, had not been assigned a position in this consensus map. Therefore, its position in the barley genome sequence [28], relative to SNPs with known linkage map positions, was used to approximate its cM position. Linkage Disequilibrium (LD) between these markers was calculated using the “Measure.R2S” function in the R package “LDcorSV.” The breeding program of each accession's origin was used to partially account for population structure for LD calculations in this panel.

An R script based on the “GWAS” function in the package rrBLUP version 4.1 [29], with minor modifications, was employed using R version 3.0.1, to conduct GWAS. Markers with a minor-allele frequency below 5% were removed. The Efficient Mixed-Model Association eXpedited (EMMAX) method, using a kinship matrix and five principal componentst, was used to account for genetic structure in this set of accessions [16]. P-values were adjusted to account for multiple comparisons using the False Discovery Rate (FDR), developed by Benjamini and Hochberg [30]. In instances where multiple closely linked markers were significant, and one of the markers was more significant than every other marker in that region for every significant trait, only the most significant marker was reported. Marker effects were based on Best Linear Unbiased Estimates (BLUEs).

Data from 2006 and 2007 were combined into a single analysis, using a fixed effect to account for differences across years, as described by Evangelou and Ioannidis [31]. This method of combining years was also used to combine barley food-quality data from an overlapping set of trials by Mohammadi et al. [20].

Positional information, Gene Ontology (GO) annotations [32], and InterPro assignments [33] were obtained for barley genes (ISBC_1.0.030312v22) through the Gramene version of the BioMart [34]. This database was scanned for genes that could be involved in the tocochromanol biosynthesis pathway, using a set of keywords to identify promising candidates. This list of genes was manually curated to remove genes that were identified by the automatic search, but after further review, were not determined to be associated with the tocochromanol biosynthesis pathway. A manual search was also conducted in which sequences from other species that are associated with the tocochromanol biosynthesis pathway were compiled from NCBI (http://www.ncbi.nlm.nih.gov/; verified 29 October 2014). For each of these sequences, the Basic Local Alignment Search Tool (BLAST) at the IPK Barley BLAST Server [35] (http://webblast.ipk-gatersleben.de/barley/; verified 29 October 2014) was used to identify regions of the barley genome homologous to these sequences of interest from other species. Annotations for barley SNPs were obtained from HarvEST (http://harvest.ucr.edu/; verified 15 December 2014).

To determine the linkage group and cM positions of candidate genes, OPA SNP markers were aligned with the barley genome sequence using the BLAST-Like Alignment Tool (BLAT) [36]. The base-pair position of SNP markers in the barley genome was determined from the BLAT output by percent identity and level of significance. The positions of candidate genes relative to their flanking markers in the genome sequence were then used to calculate approximate cM positions.

## Results

### Phenotypic data

**Fig 1:**
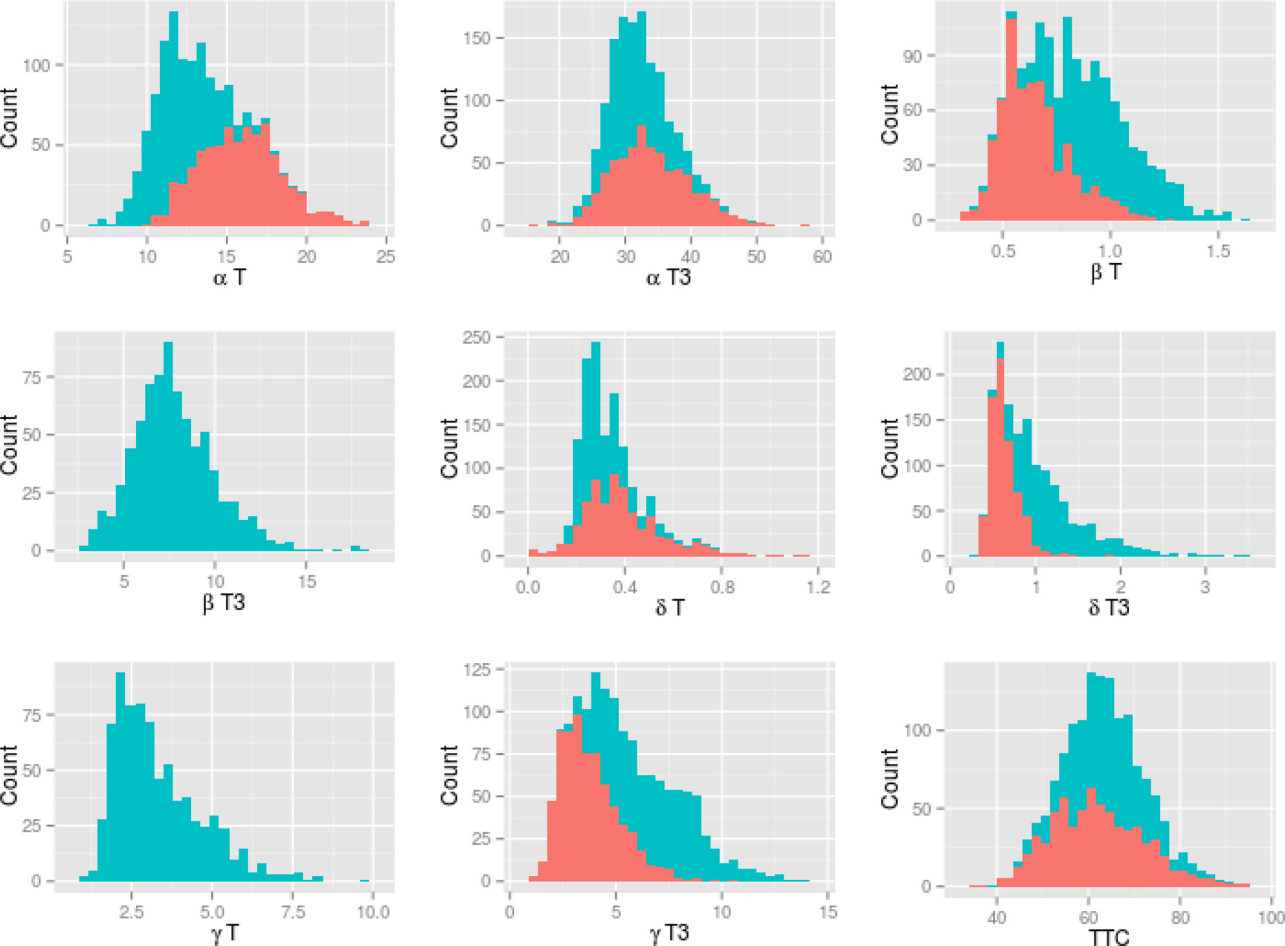
Distributions of concentrations of all tocochromanol forms and Total Tocochromanol (TTC). Reliable data for βT3 and γT are not available for 2006. Red represents 2006 and blue represents 2007.

There were detectable concentrations of all tocochromanols in all accessions in both years (Table 1; Fig 1; S1 and S2 Figs). Including both years, αT concentrations ranged from 6.8 mg/kg to 23.9 mg/kg and total tocochromanol (TTC) concentrations ranged from 30.9 mg/kg to 94.1 mg/kg. Considering all forms, αT3 had the highest average concentration, and δT had the lowest average concentration. Means and standard errors for all tocochromanol forms are presented in Table 1. An analysis of variance showed that both year and breeding program had significant effects on αT and TTC concentrations (S1 Table). Row-type had a significant effect on both αT and TTC (S1 Table). αT, αT3 and δT concentrations were higher in 2006 (irrigated) than in 2007 (dryland), whereas the reverse was true for βT, δT3 and γT3 and TTC (p<0.0001 for all comparisons). As noted in the Materials and Methods, βT3 and γT were not distinguished in the analysis of 2006 samples. Therefore, it is not possible to assess the effect of year/management practice on these forms. Which breeding program had germplasm with the highest average tocochromanol concentration varied by year and tocochromanol form. For example, in 2006, germplasm from MT had the highest average concentration of αT and TTC whereas in 2007, germplasm from UT had the highest average concentration of αT and germplasm from USDA-ARS-Idaho had the highest average concentration of TTC. In both years, germplasm from the MN had the lowest average concentration of both αT and TTC.

**Table 1:** Means and standard errors for concentrations (mg/kg) of all tocochromanol forms and Total Tocochromanol (TTC) for accessions from each of the eight breeding programs, separated by year.

Program	Year	αT	αT3	βT	βT3	δT	δT3	γT	γT3	TTC
AB	2006	15.84±0.19	34.63±0.55	0.72±0.01	-	0.32±0.01	0.64±0.02	-	3.30±0.11	61.68±0.83
AB	2007	12.86±0.18	32.68±0.58	1.01±0.02	8.23±0.23	0.35±0.01	1.37±0.05	3.92±0.16	7.07±0.17	67.48±0.87
BA	2006	16.26±0.25	33.89±0.47	0.70±0.02	-	0.43±0.01	0.67±0.02	-	4.42±0.17	63.96±0.95
BA	2007	12.18±0.16	31.64±0.36	0.97±0.02	8.51±0.22	0.30±0.01	1.40±0.04	3.46±0.13	7.00±0.21	65.45±0.63
MN	2006	13.30±0.18	30.95±0.49	0.65±0.02	-	0.36±0.01	0.67±0.01	-	2.64±0.08	53.28±0.77
MN	2007	10.83±0.10	31.94±0.32	0.84±0.02	6.66±0.13	0.33±0.01	1.04±0.03	2.46±0.07	4.48±0.09	58.59±0.50
MT	2006	19.12±0.18	40.46±0.52	0.72±0.02	-	0.49±0.02	0.60±0.01	-	4.87±0.13	75.92±0.79
MT	2007	11.61±0.20	29.04±0.33	0.82±0.01	8.24±0.29	0.26±0.01	1.43±0.07	3.18±0.09	9.14±0.18	63.72±0.71
N2	2006	16.99±0.19	35.78±0.56	0.63±0.02	-	0.36±0.01	0.52±0.01	-	3.61±0.11	64.23±0.73
N2	2007	11.09±0.12	30.87±0.37	1.13±0.02	7.68±0.24	0.28±0.01	1.16±0.03	2.97±0.09	7.09±0.14	62.28±0.74
N6	2006	15.71±0.14	33.82±0.53	0.57±0.01	-	0.27±0.01	0.67±0.01	-	3.13±0.08	58.87±0.76
N6	2007	11.48±0.17	33.64±0.44	1.09±0.02	7.34±0.18	0.27±0.01	1.06±0.03	2.24±0.06	4.94±0.10	62.06±0.75
UT	2006	15.84±0.42	28.95±0.98	0.63±0.03	-	0.47±0.03	0.69±0.05	-	3.91±0.29	56.90±1.56
UT	2007	14.08±0.22	33.28±0.52	0.88±0.02	6.97±0.21	0.34±0.01	1.09±0.05	3.68±0.12	7.01±0.19	67.34±0.93
WA	2006	13.95±0.17	30.21±0.4	0.58±0.010	-	0.49±0.02	0.62±0.02	-	4.36±0.15	59.11±0.87
WA	2007	12.17±0.14	30.82±0.31	0.87±0.02	8.83±0.22	0.38±0.01	1.28±0.04	4.74±0.15	7.12±0.16	66.21±0.73
Mean	2006	15.89±0.10	33.98±0.22	0.65±0.01	-	0.39±0.01	0.63±0.01	-	3.76±0.05	62.13±0.39
Mean	2007	12.04±0.07	31.73±0.15	0.95±0.01	7.81±0.08	0.31±0.00	1.23±0.02	3.34±0.05	6.74±0.08	64.15±0.28

### GWAS and marker-trait associations

**Fig 2:**
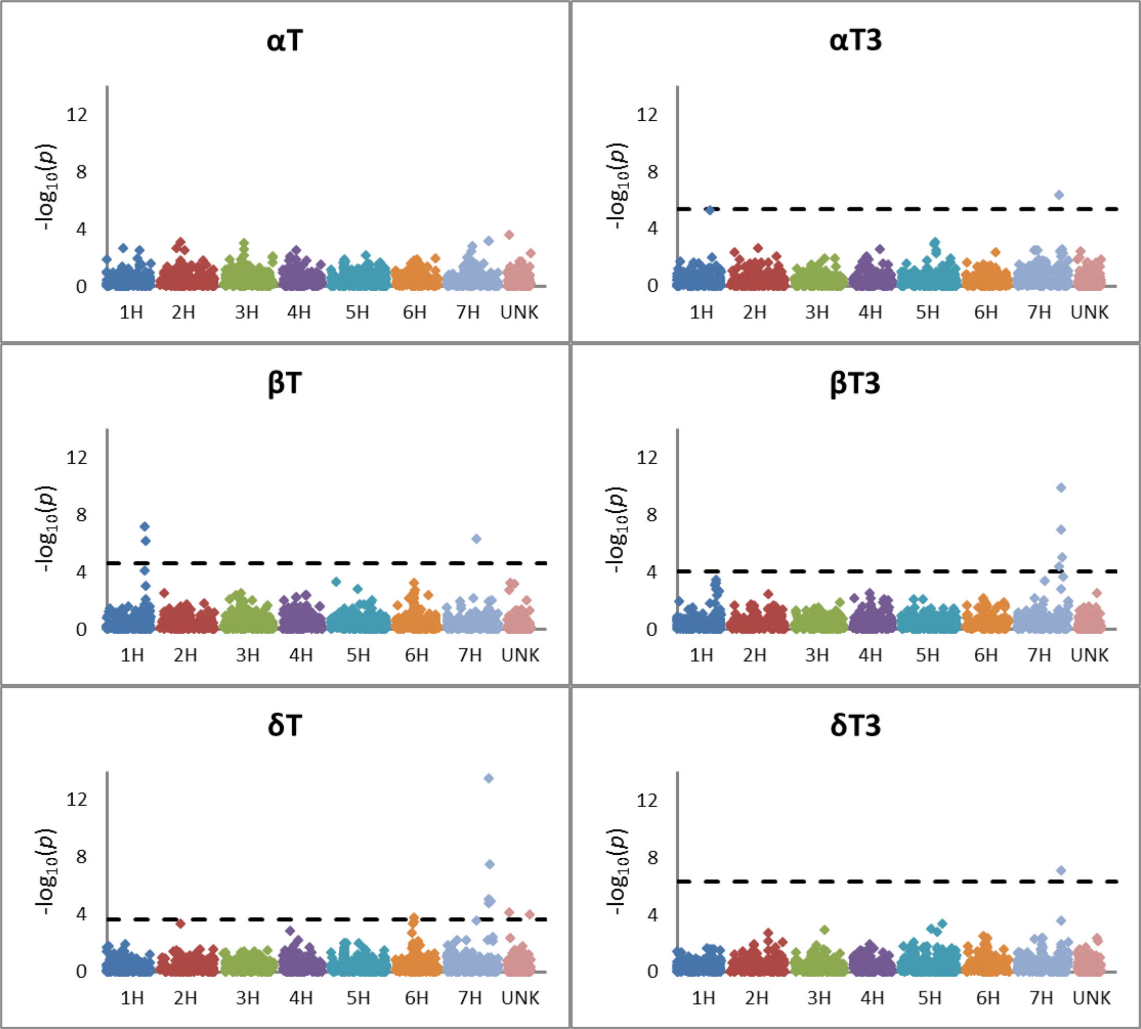
Manhattan plots showing results of GWAS for concentrations of αT, αT3, βT, βT3, δT, and δT3. In analyses where one or more markers met the significance threshold determined by a false-discovery rate adjustment, a dotted line shows the significance threshold. Points in pink, adjacent to chromosome 7H, represent unmapped markers.

**Fig 3:**
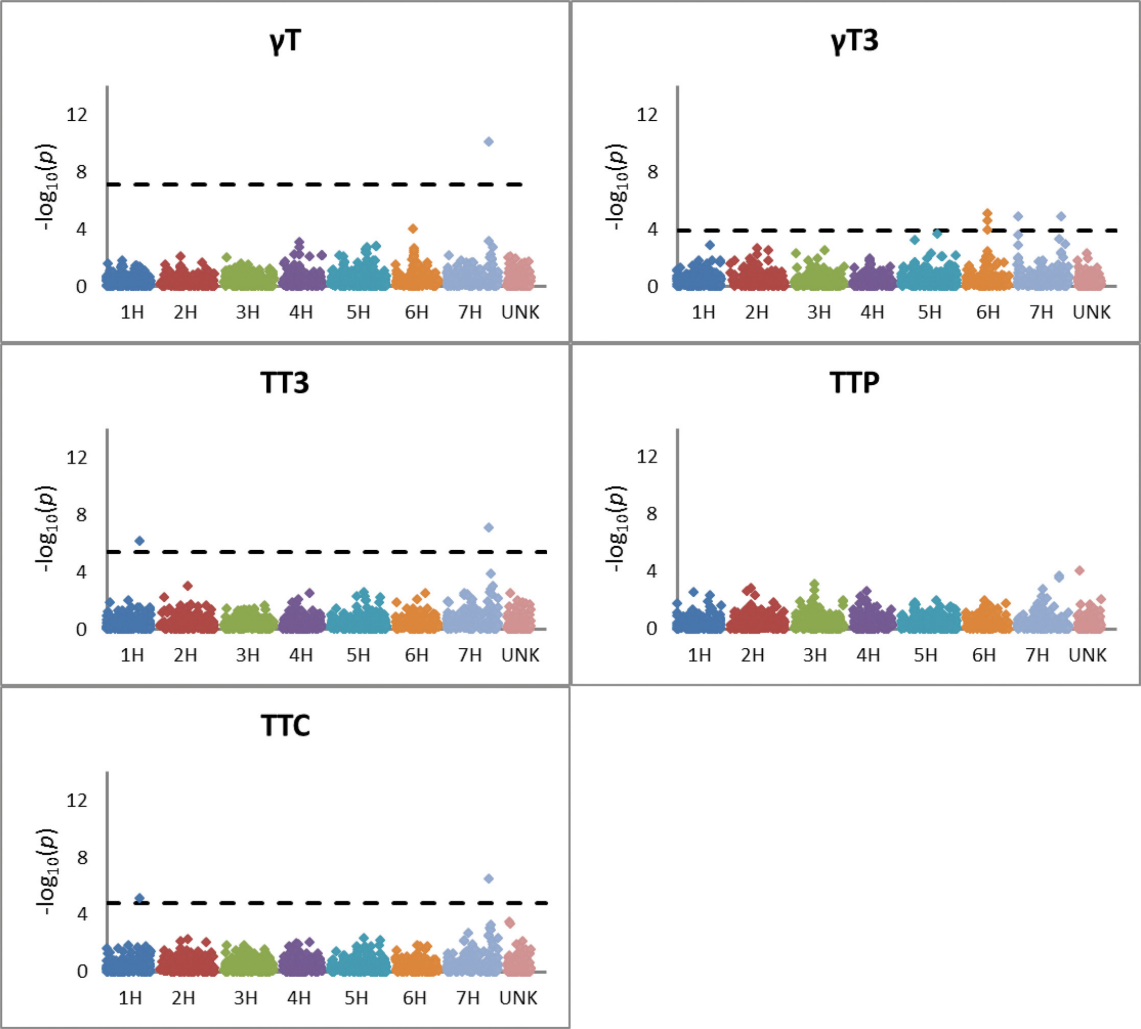
Manhattan plots showing results of GWAS for concentrations of γT, γT3, TT3, TTP, and TTC. In analyses where one or more markers met the significance threshold determined by a false-discovery rate adjustment, a dotted line shows the significance threshold. Points in pink, adjacent to chromosome 7H, represent unmapped markers.

Q-Q plots indicate that the model with five principal components adequately accounted for population structure, thereby controlling false positives (S3 Fig). Principal component analysis identified row-type and breeding program as major drivers of structure in this set of germplasm (S4 Fig). The proportions of the genotypic variation explained by the first five principal components were 0.316, 0.055, 0.040, 0.028, and 0.022. Thirteen SNP markers were significantly associated with one or more of the tocochromanol forms and/or fractions (Table 2; Figs 2 and 3). The two significant SNPs on chromosome 1H were at cM 110 (associated with total tocotrienol (TT3) and TTC), and the second at cM 128 (associated with βT). Based on the linkage distance between these SNPs and on an analysis of linkage disequilibrium (S1 File), these are two distinct regions with different QTLs/candidate genes and are described as 1H-A and 1H-B (Table 3). On chromosome 6H, two SNPs were significant: one at cM 58 (associated with δT), and one at cM 71 (associated with γT3). These two regions are described as 6H-A and 6H-B. The remaining eight SNPs were on 7H and formed three regions (7H-A, 7H-B, and 7H-C): one at cM 1 (associated with γT3); two at cM interval 95–96 (associated with βT and δT); and five at cM interval 136–145 (associated with αT3, βT3, δT, δT3, γT, γT3, TT3, and TTC). There were no significant associations of any mapped SNPs with αT or total tocopherol (TTP).

**Table 2:** Significance (-log_10_(*p*)) of markers for concentration of all tocochromanol forms fractions, and Total Tocochromanol (TTC).

Marker	Chromosome	Position	αT	αT3	βT	βT3	δT	δT3	γT	γT3	TT3	TTP	TTC
11_20021	1H	110	2.47	5.31	0.33	0.19	0.07	1.60	0.67	2.88	**6.17**	2.38	**5.16**
11_10586	1H	128	0.13	0.26	**7.17**	3.03	0.63	0.16	0.63	0.06	0.21	0.06	0.13
12_30802	6H	58	1.26	0.42	1.82	0.23	**3.77**	0.23	2.63	0.45	0.57	1.60	0.23
12_30637	6H	71	0.08	0.09	0.04	0.40	0.70	0.99	0.72	**5.09**	0.75	0.10	0.97
12_30296	7H	1	0.20	0.91	0.20	0.52	0.64	1.26	0.14	**4.83**	1.89	0.17	1.27
11_21201	7H	95	1.51	0.34	**6.26**	0.22	3.56	0.25	0.37	0.02	0.21	2.13	0.97
11_20311	7H	96	3.60	1.99	2.73	0.01	**4.16**	0.41	1.53	0.38	1.58	4.10	3.50
11_21209	7H	136	0.35	1.33	1.42	**4.39**	**13.53**	1.21	**10.05**	0.06	1.20	0.73	2.48
11_10861	7H	139	3.13	**6.37**	1.95	1.34	**5.06**	0.90	0.35	3.29	**7.04**	3.69	**6.52**
11_10797	7H	141	0.22	2.43	0.14	1.11	**7.50**	0.17	1.97	0.95	2.54	0.38	3.08
12_10973	7H	142	0.66	2.07	0.46	**6.90**	**4.91**	3.57	2.24	2.25	2.79	0.42	2.91
11_10885	7H	145	0.25	2.32	0.30	**9.84**	0.08	**7.06**	0.07	**4.84**	3.87	0.24	3.28
12_31511	Unknown	Unknown	0.77	0.02	0.10	0.14	**4.00**	0.32	1.01	0.34	0.01	0.86	0.18

Bolded values show significant marker-trait associations.

**Table 3:** Significant SNPs associated with tocochromanols, and annotated sequences known or predicted to be associated with the tocochromanol biosynthesis pathway that occurred within 2 cM of a significant marker.

SNP Marker orAnnotated Genome Sequence	Chromosome/ Region*	Position (cM)**	Position (bp)***	Morex Contig Number	Sequence Annotation
MLOC_16149	1H-A	108	430,628,647–430,636,996	157254	Resembles genes encoding *HGGT* and *VTE2*, and enzymes upstream of geranylgeranyl diphosphate biosynthesis
11_20021	1H-A	110	Unknown	48282	SNP Marker
11_10586	1H-B	128	447,413,118	171284	SNP Marker
MLOC_72891	6H-A	56	84,648,047–84,652,085	62562	Resembles genes encoding enzymes upstream of geranylgeranyl diphosphate biosynthesis
MLOC_44750	6HA	58	145,761,890–145,768,330	275292	Resembles genes encoding enzymes upstream of geranylgeranyl diphosphate biosynthesis
12_30802	6H-A	58	164,529,448	1592014	SNP Marker
MLOC_66290	6H-A	59	265,678,548–265,680,735	51352	Resembles genes encoding enzymes upstream of geranylgeranyl diphosphate biosynthesis
12_30637	6H-B	71	432,652,993	1559740	SNP Marker
MLOC_13082	6H-B	71	437,175,681–437,178,395	1564754	Resembles gene encoding *VTE4*
12_30296	7H-A	1	2,219,656	178733	SNP Marker
11_21201	7H-B	95	Unknown	45924	SNP Marker
11_20311	7H-B	96	Unknown	1566790	SNP Marker
11_21209	7H-C	136	570,924,522	1579096	SNP Marker
MLOC_37476	7H-C	138	575,423,330–575,428,610	25484480	Resembles genes encoding enzymes upstream of geranylgeranyl diphosphate, and *VTE2-1*
11_10861	7H-C	139	Unknown	7405	SNP Marker
11_10797	7H-C	141	577,196,196	354235	SNP Marker
12_10973	7H-C	142	578,753,062	77635	SNP Marker
MLOC_12567	7H-C	145	584,322,177–584,325,723	1563577	*HGGT*
11_10885	7H-C	145	584,352,965	2547604	SNP Marker

*Regions, as defined in text, refer to chromosome regions with different QTLs/candidate genes. **Linkage map positions (Muñoz-Amatriaín et al. 2011).

***Genome sequence positions, (International Barley Genome Sequencing Consortium 2012).

In region 7H-B the two significant markers are in linkage disequilibrium, but the two markers were significant for different tocochromanol forms. In region 7H-C, in some cases adjacent significant markers were in linkage disequilibrium. However, the middle significant marker was not in linkage disequilibrium with either the first or last significant marker, providing some evidence for multiple QTL in this region. There were no significant associations of SNPs with αT or total tocopherol (TTP).

### Candidate genes

None of the significant markers were within an Expressed Sequence Tag (EST) that was annotated within HarvEST as being potentially related to tocochromanol biosynthesis. Of the thirteen significant markers, seven were within 2 cM of at least one sequence homologous with genes known to be associated with the tocochromanol biosynthesis pathway in barley and/or other plants (Table 3). On 1H, candidate gene MLOC_16149, with sequence homology to *VTE2* (as described by Collakova and DellaPenna [37]), homogentisate geranylgeranyltransferase (as described by Cahoon et al. [14]), and multiple enzymes upstream of geranylgeranyl diphosphate biosynthesis (a precursor to all tocochromanols; Cahoon et al. [14]), including farnesyl diphosphate synthase (as described by Matsushita et al. [38]) and homogentisate farnesyltransferase (as described by Sadre et al. [39]), was identified at cM 108–2 cM from marker 11_20021. No candidate genes were identified within 2 cM of marker 11_10586.

On 6H, three candidate genes were identified within 2 cM of marker 12_30802: MLOC_72891, MLOC_44750, and MLOC_66290. Each of these candidate genes has sequence homology to multiple enzymes upstream of geranylgeranyl diphosphate biosynthesis. On 6H at cM 71, the candidate gene MLOC_13082, with sequence homology to enzyme *VTE4* (as described by Shintani and DellaPenna [40]) was 0 cM from marker 12_30637. On 7H, no candidate genes were identified within 2 cM of markers 12_30296, 11_21201, or 11_20311. Two candidate genes were identified within 2 cM of the group of markers in cM interval 136–150: MLOC_12567 encoding *HG*GT, and MLOC_37476 with sequence homology to *VTE2* and multiple enzymes upstream of geranylgeranyl diphosphate biosynthesis.

### Allele effects and distributions

As shown in Table 4, the best linear unbiased estimators (BLUEs) for allele effects reveal substantial phenotypic variation associated with allele substitutions at the significant SNPs. Both alleles at each significant SNP were present in most breeding programs (S2 Table). The accessions from the USDA-ARS- ID program and UT had the highest levels of allelic diversity, never having less than 9% and 6% of the minor allele, respectively.

**Table 4:** Best Linear Unbiased Estimators (BLUEs) for concentration (mg/kg) of all tocochromanol forms, fractions, and Total Tocochromanol (TTC).

Marker	Chromosome	Position	αT	αT3	βT	βT3	δT	δT3	γT	γT3	TT3	TTP	TTC
11_20021	1H	110	0.50	1.83	0.010	0.09	0.002	0.062	-0.12	0.36	**2.26**	0.51	**3.02**
11_10586	1H	128	-0.04	-0.16	**0.047**	0.44	0.008	-0.007	0.08	-0.01	-0.15	0.02	0.15
12_30802	6H	58	-0.38	0.42	-0.038	0.13	**-0.045**	0.018	-0.36	0.12	0.61	-0.46	-0.44
12_30637	6H	71	0.03	0.10	0.002	0.17	0.013	0.045	0.13	**0.50**	0.63	0.04	1.09
12_30296	7H	1	-0.07	0.57	0.006	0.19	-0.011	0.048	0.03	**0.44**	1.05	-0.07	1.19
11_21201	7H	95	-0.28	-0.24	**-0.053**	0.08	-0.029	0.013	-0.06	0.00	-0.18	-0.36	-0.84
11_21209	7H	136	0.08	0.53	0.018	**0.58**	**0.050**	0.034	**0.45**	0.01	0.56	0.15	1.30
11_10861	7H	139	-0.33	**-1.18**	-0.020	-0.26	**-0.026**	-0.024	-0.05	-0.22	-1.42	-0.38	**-1.99**
11_10797	7H	141	-0.07	-0.91	-0.004	-0.28	**-0.043**	-0.009	-0.20	-0.14	-1.06	-0.11	-1.74
12_10973	7H	142	-0.18	0.90	0.011	**0.91**	**0.038**	0.084	0.24	0.26	1.22	-0.13	1.84
11_10885	7H	145	0.07	-0.76	-0.006	**-0.85**	0.002	**-0.097**	0.01	**-0.32**	-1.17	0.06	-1.56
11_20311	7H	95	0.62	1.04	0.043	0.01	**0.041**	-0.024	0.24	0.09	1.03	0.70	2.44
12_31511	Unknown	Unknown	0.19	0.02	-0.003	-0.06	**0.032**	-0.016	0.15	-0.07	-0.01	0.21	0.24

Positive values indicate that individuals with the “A” allele has a higher tocochromanol concentration, and negative values indicates that genotypes with the “B” allele have a higher tocochromanol concentration. Bolded values show significant marker-trait associations.

## Discussion

### Phenotypic variation for tocochromanols

The sample of 1,466 elite accessions that we analyzed followed the same trends reported in other barley studies [15] in terms of the relative concentrations of specific tocochromanol forms, with αT3 generally being highest, and δT generally being the lowest. In general, the concentrations of tocochromanol forms in this study were comparable to previous studies [15,41]. The highest αT concentration observed was 1.72 times higher than the average αT concentration, and the highest TTC concentration observed was 1.49 times higher than the average TTC concentration.

Differences were observed in tocochromanol concentrations over the two years of this study, although this was confounded by the different germplasm arrays grown each year (Table 1; Fig 1; S1 and S2 Figs). While the 2006 growing season in Bozeman, Montana was relatively typical, the 2007 growing season was characterized by extremes, with 18.5 cm of snowfall recorded on May 29^th^, followed by a July that was possibly the hottest on record, and had little precipitation (National Weather Service; http://nws.noaa.gov/climate/local_data.php?wfo=tfx; verified 3 November 2014). Irrigation in the 2006 growing season, but not in the 2007 growing season, resulted in differential moisture stress in the two years. Oliver et al. [15] also reported that moisture availability and temperature are important environmental factors associated with tocochromanol concentrations. Future experiments, in which barley varieties are replicated, and a range of environmental factors are controlled, would help to better understand the effect of specific environmental factors on tocochromanol concentrations, as well as investigate a possible genotype by environment factor. Seed of this barley GWAS panel is available and, together with the available genotype data, would be a useful starting point for such experiments.

Given the observed values for tocochromanol forms, a key question is whether barley can be a viable source of these compounds for human nutrition. The answer to this question is complicated by the fact that a RDA has only been established for αT, which is 15 mg/day for adults (National Institute of Health Office of Dietary Supplements; http://ods.od.nih.gov/factsheets/VitaminE-HealthProfessional/; verified 29 October 2014). Using the accession with the highest αT concentration, 06MT-55 with 23.9 mg/kg αT, a healthy adult would need to consume approximately 628 g of barley (dry weight) per day to meet their RDA. Therefore, it is not realistic to imagine barley as a *sole or principal* source of αT in human diets: other plant products are superior sources of αT. Sunflower seeds, for example contain approximately 351.7 mg/kg of αT (USDA-ARS National Nutrient Database; http://ndb.nal.usda.gov/ndb/foods/show/3658; verified 29 October 2014).

Tocochromanols other than αT are also reputed to provide nutritional benefits. γT, for example, is superior to αT in detoxifying reactive nitrogen species [42], an important consideration in chronic inflammation, and for smokers or individuals subject to air pollution. Furthermore, in cellular assays, γT was shown to provide neuroprotective effects at concentrations 4 to 10 fold lower than typically found in human plasma [43]. Diets rich in tocotrienols have been shown to reduce cholesterolgenesis in chicks [44] and in humans [45]. Tocopherols do not exhibit this property.

Whole grain barley, however, brings valuable components to human diets in addition to tocochromanols, including β-glucan [46,47]. Therefore, the focus for breeding food barley should more realistically be on the total nutritional composition, and not exclusively on tocochromanol content.

An important question to address in this context is the role of tocochromanols in barley growth, development, and reproductive fitness. Studies with *Arabidopsis* mutants deficient in tocopherol biosynthesis clearly illustrate a role for these metabolites in cold tolerance [48] and these mutants were employed to demonstrate a critical role for tocopherols in germination and seed storage [49]. In monocots, a correlation between γT concentration and enhanced germination and root growth has been shown in barley [50], and in emmer, seeds collected from a location with higher abiotic stresses had higher tocochromanol concentrations than those collected from locations with lower abiotic stresses [51]. Given the available phenotype and genotype data, and reserve seed, the GWAS panel used for this study could be used, in future analyses and experiments, to compare the genome locations of tocochromanol genes/QTLs in relationship to genes/QTLs related to productivity and stress resistance. Coincident genes/QTLs would be justification to proceed with testing hypotheses regarding pleiotropic effects of tocochromanol concentration on plant health and productivity.

### QTL and candidate genes

Thirteen significant SNP-tocochromanol trait associations were detected on three chromosomes (1H, 6H, and 7H) (Table 1). The significant SNPs within each linkage group can be further subdivided into seven discrete genomic regions, based on the large linkage distances separating groups of significant markers (Table 3) and an analysis of linkage disequilibrium (S1 File). In five of these regions, there are candidate genes (1H-A, 1H-B, 6H-A, 7H-B and 7H-C) based on annotation: *HGGT*, *VTE1*, *VTE2*, *VTE4*, and a sequence with similarity to a gene encoding geranylgeranyl diphosphate synthase. In the remaining two regions (6H-B and 7H-A) there are significant QTL: marker associations without candidate genes. Possible explanations include (i) there are structural genes in these regions involved in tocochromanol synthesis but they are as yet undetected due to gaps in the genome sequence, and/or (ii) the presence of regulatory elements with functions in the tocochromanol pathway. Alignment of the barley consensus map and the map used by Oliver et al. [15] reveals some, but not complete, overlap with significant markers we detected (S2 File). That our findings are consistent with those of Oliver et al. [15] confirms that GWAS and bi-parental QTL mapping can be effective in the dissection of complex traits, given adequate population sizes, robust phenotyping and reasonable marker density. An advantage of GWAS is that a panel can be assembled immediately, whereas with a biparental population, for self-pollinated crops, it can take several years after an initial cross to achieve the amount of seed and the desired level of homogeneity before phenotypic evaluations can begin [52].

GWAS can provide fundamental insights into the genetic basis of economically important traits, as evidenced by recent reports in a range of crop plants, including barley [19–21]. By providing estimates of the number and genomic context of sequences affecting target traits in relevant germplasm, GWAS can also provide targets for Marker Assisted Selection (MAS) that will increase the efficiency of development for superior varieties. The panel used in this GWAS could be used to quickly generate near-isogenic lines for QTL by taking advantage of heterogenous inbred families [53]. The Barley CAP germplasm used in this study was previously used to identify QTL for disease resistance [54], and subsequent lines from the panel that were heterozygous at QTL were used to develop sets of near-isogenic lines to validate those QTL [55]. Near-isogenic line pairs developed from these lines could be used to study environmental effects, refine map positions, identify multiple alleles at QTL, or investigate QTL interaction with genetic background.

In terms of future breeding applications, two accessions from AB (2AB04-01084-6 and 2AB04-01084-15) have the favorable alleles at each of the 13 SNPs significantly associated with one or more tocochromanol forms and/or fractions. These two accessions may be a valuable resource for developing varieties with enhanced tocochromanol concentrations. The TTC concentrations of these lines (81.79 mg/kg and 80.73 mg/kg, respectively) is higher than the average accession in 2007, the year that these lines were grown, but lower than the highest accession grown in that year (6B05-0788, from BA), which had a TTC of 90.02 mg/kg. The αT concentrations for these accessions, 17.51 mg/kg and 14.29 mg/kg, were also higher than the average accession in 2007, but lower than the highest accession grown in that year (MT050165 from MT), which had an αT concentration of 23.88 mg/kg. In this set of germplasm, no accession had all negative alleles at the 13 significant SNPs.

## Conclusions

This study demonstrates that GWAS can detect genetic determinants of complex traits in a panel of elite germplasm. This approach to QTL and candidate gene identification can complement the use of bi-parental mapping populations specifically tailored to each trait. A total of 13 marker-trait associations for tocochromanol concentrations in barley were identified. The significant SNPs were found in seven genomic regions on three chromosomes. Five of the seven associations were with markers near genes associated with the tocochromanol pathway. The availability of the draft of the barley genome sequence, published by the International Barley Genome Sequencing Consortium [28], enabled the alignment of QTLs with candidate genes. This information will be useful in future studies directed at understanding the role(s) of tocochromanols in barley growth, development, stress resistance and productivity. It will also be useful in breeding food barley varieties that can supply moderate amounts of tocochromanols in human diets within a framework of whole grain nutrition.

### Disclaimer

Mention of trade names or commercial products in this publication is solely for the purpose of providing specific information and does not imply recommendation or endorsement by the U.S. Department of Agriculture. The USDA is an equal opportunity provider and employer.

## Supporting Information

S1 Fig Distributions of αT across breeding programs. (DOC)

S2 Fig Distributions of TTC across breeding programs. (DOC)

S3 Fig Q-Q plots showing the distribution of p-values, plotted against the expected distribution of p-values, for each analysis in this study. (DOC)

S4 Fig Principal component analysis of all three combinations of the first, second, and third principal components for this set of germplasm. (DOC)

S1 File Analysis of linkage disequilibrium between genetic markers in regions of the barley genome identified as significant in this study. (PPT)

S2 File Comparison of the genetic maps used in this study, and by Oliver et al. (2014). (PPT)

S1 Table Analyses of variances for α-tocopherol and TTC concentration in barley. (DOC)

S2 Table Distribution of alleles for significant markers associated for tocochromanols for each of eight breeding program. (DOC)
