# Supplementary figures and images for: Quantitative Trait Loci Associated with the Tocochromanol (Vitamin E) Pathway in Barley
Source: PLoS One. 2015 Jul 24;10(7):e0133767. doi: 10.1371/journal.pone.0133767 (PMC4514886; doi:10.1371/journal.pone.0133767)

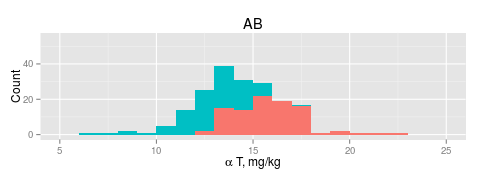

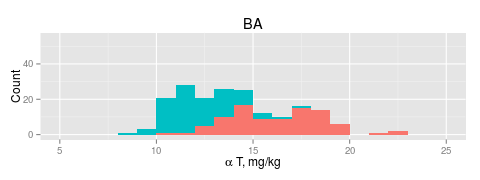

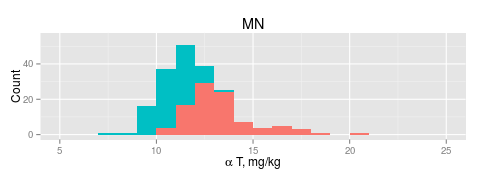

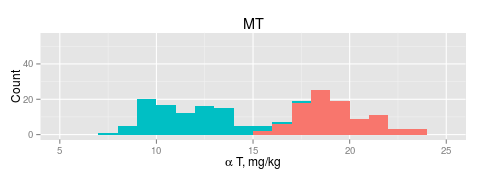

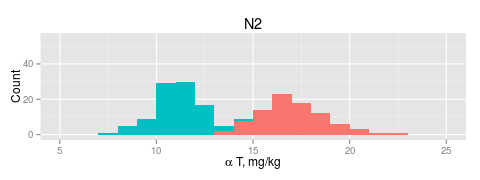

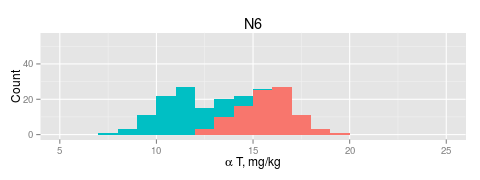

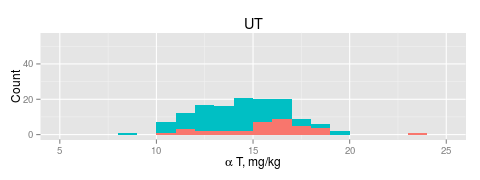

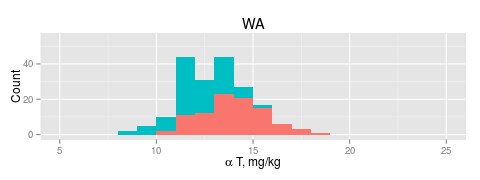


**S1 Fig. Distributions of αT across breeding programs.** Red represents 2006 and blue represents 2007.

Supplement: S1 Fig — (DOC) [file pone.0133767.s001.doc]

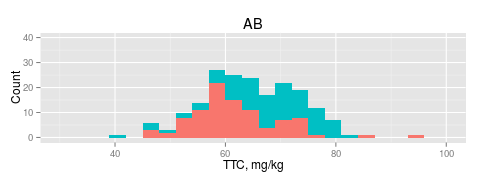

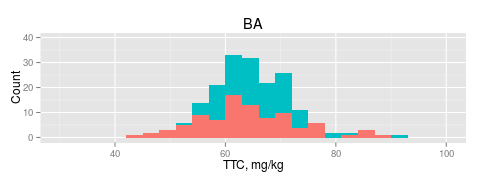

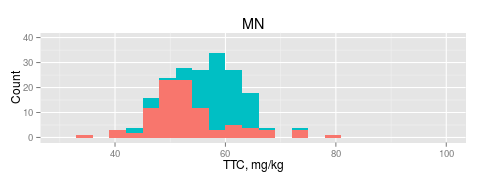

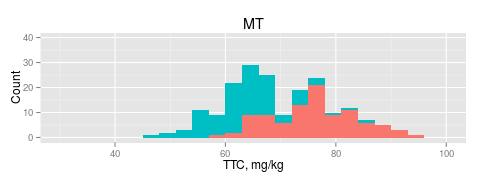

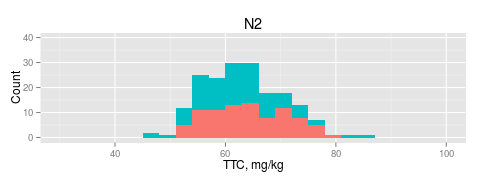

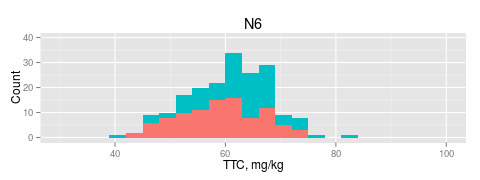

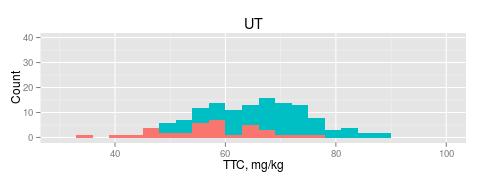


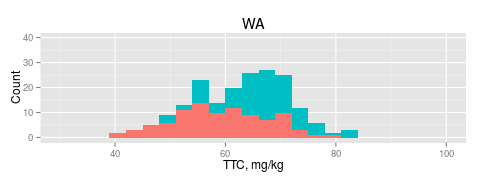


**S2 Fig. Distributions of TTC across breeding programs.** Red represents 2006 and blue represents 2007.

Supplement: S2 Fig — (DOC) [file pone.0133767.s002.doc]
