# Supplementary material for: Quantitative Trait Loci Associated with the Tocochromanol (Vitamin E) Pathway in Barley
Source: PLoS One. 2015 Jul 24;10(7):e0133767. doi: 10.1371/journal.pone.0133767 (PMC4514886; doi:10.1371/journal.pone.0133767)
Supplement: S3 Fig — (DOC) [file pone.0133767.s003.doc]

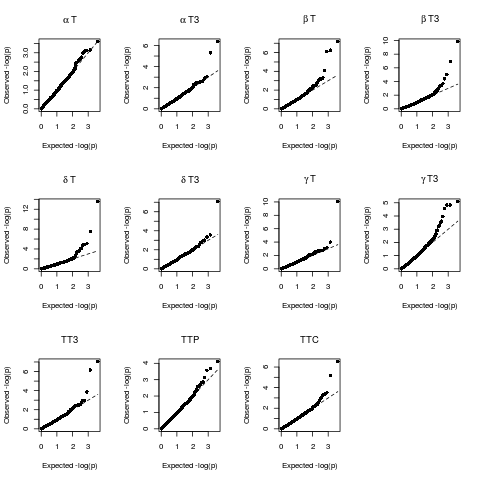


**S3 Fig. Q-Q plots showing the distribution of p-values, plotted against the expected distribution of p-values, for each analysis in this study.**
