# Supplementary material for: Quantitative Trait Loci Associated with the Tocochromanol (Vitamin E) Pathway in Barley
Source: PLoS One. 2015 Jul 24;10(7):e0133767. doi: 10.1371/journal.pone.0133767 (PMC4514886; doi:10.1371/journal.pone.0133767)
Supplement: S4 Fig — (DOC) [file pone.0133767.s004.doc]

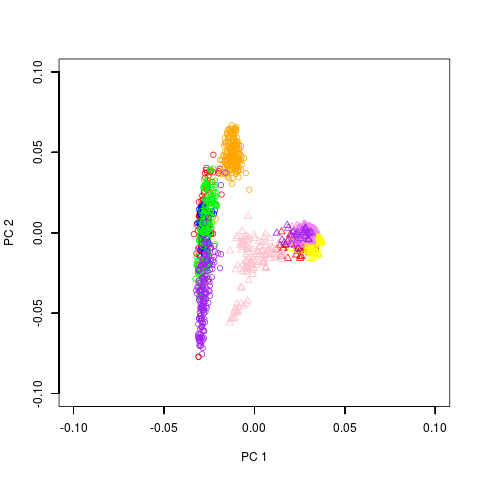

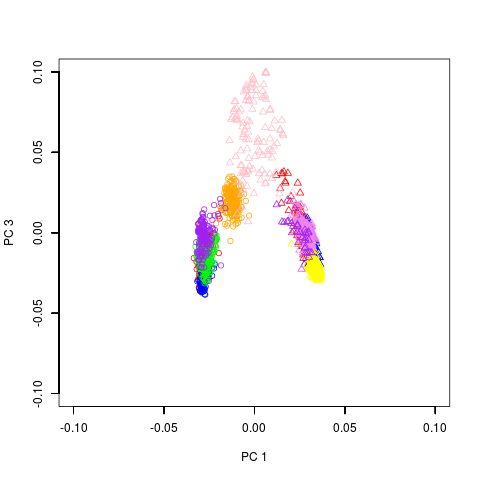

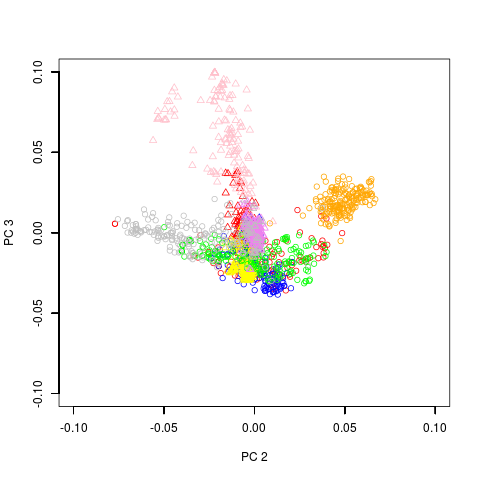


**S4 Fig. Principal component analysis of all three combinations of the first, second, and third principal components for this set of germplasm.** Two-row accessions are depicted by circles, and six-row accessions are depicted by triangles. The program of origin for each accessions is color-coded, as follows: AB (red), BA (blue), the MN (yellow), MT (green), N2 (orange), N6 (violet), UT (pink), and WA (grey).
