# Supplementary material for: Quantitative Trait Loci Associated with the Tocochromanol (Vitamin E) Pathway in Barley
Source: PLoS One. 2015 Jul 24;10(7):e0133767. doi: 10.1371/journal.pone.0133767 (PMC4514886; doi:10.1371/journal.pone.0133767)
Supplement: S1 File — (PPT) [file pone.0133767.s005.ppt]

## Slide 1
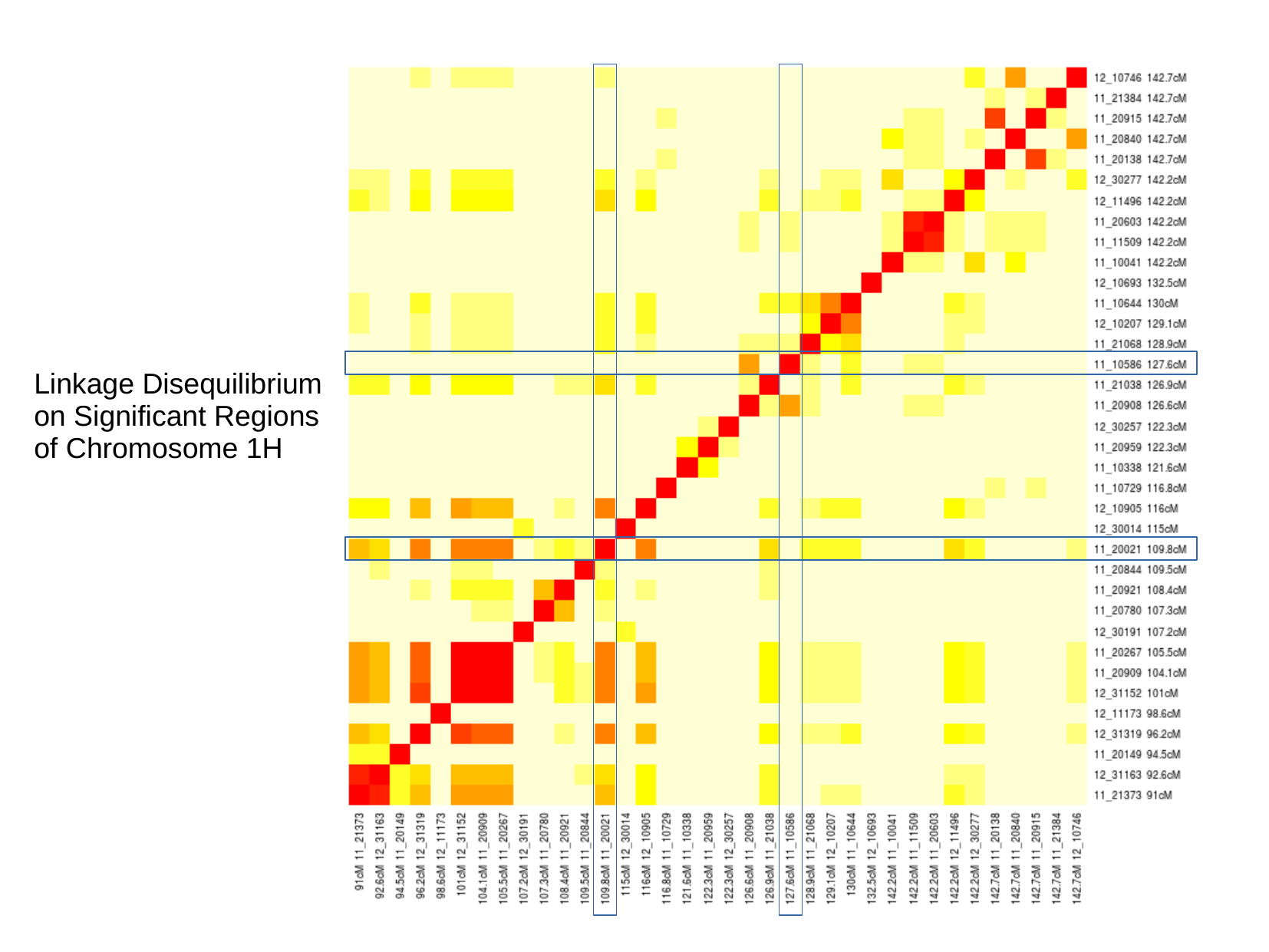

Linkage Disequilibrium on Significant Regions of Chromosome 1H

## Slide 2
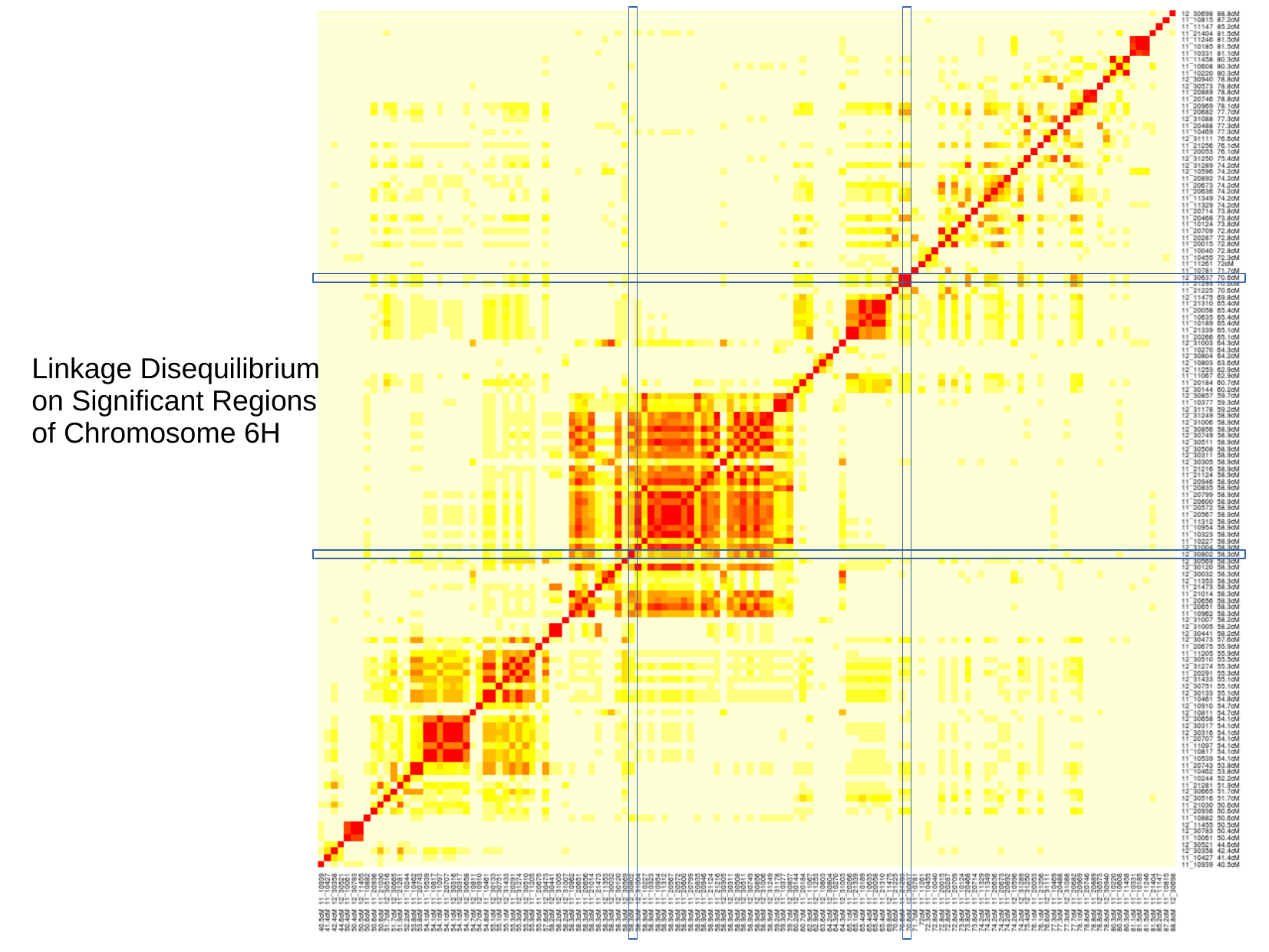

Linkage Disequilibrium on Significant Regions of Chromosome 6H

## Slide 3
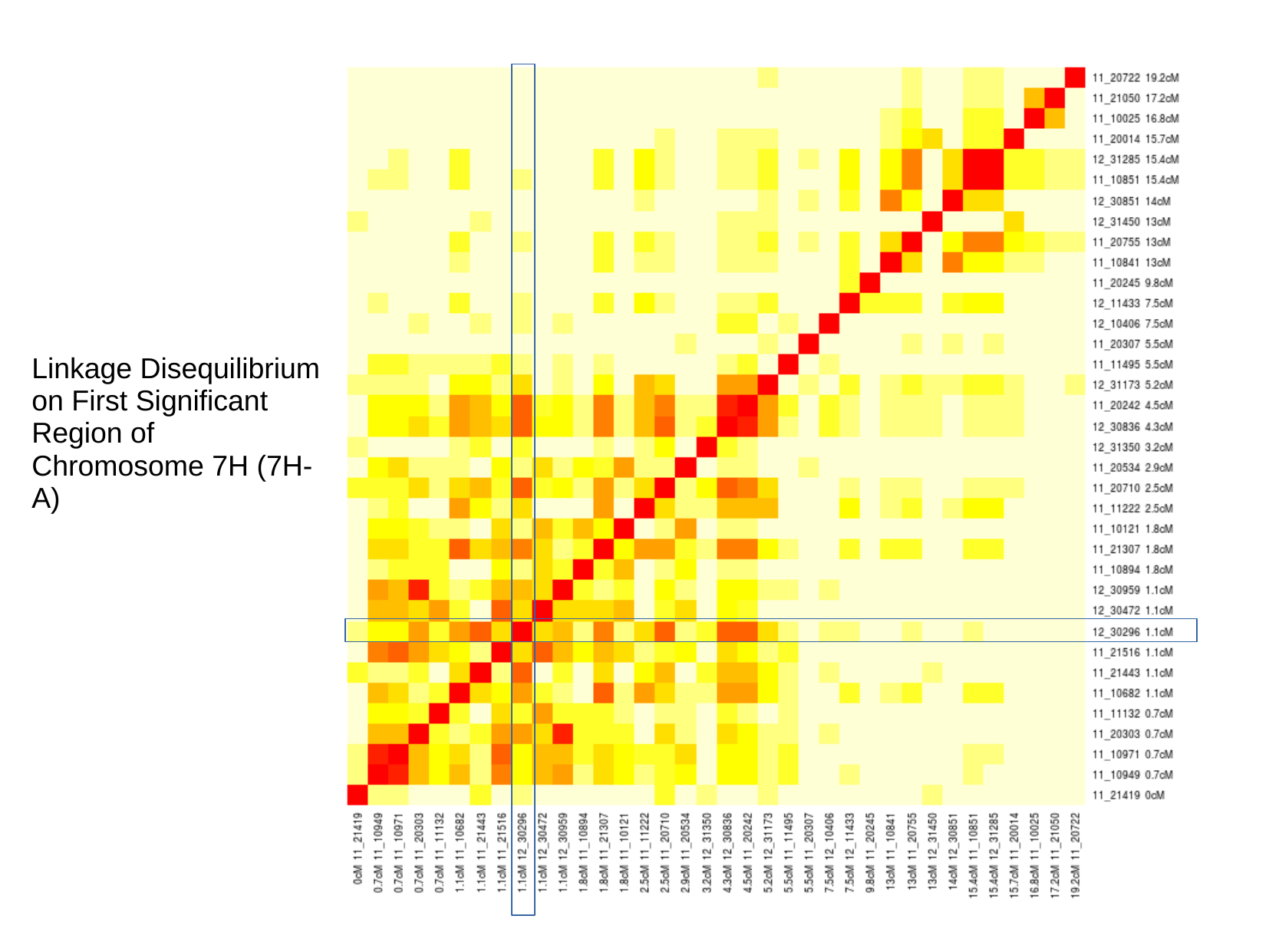

Linkage Disequilibrium on First Significant Region of Chromosome 7H (7H-A)

## Slide 4
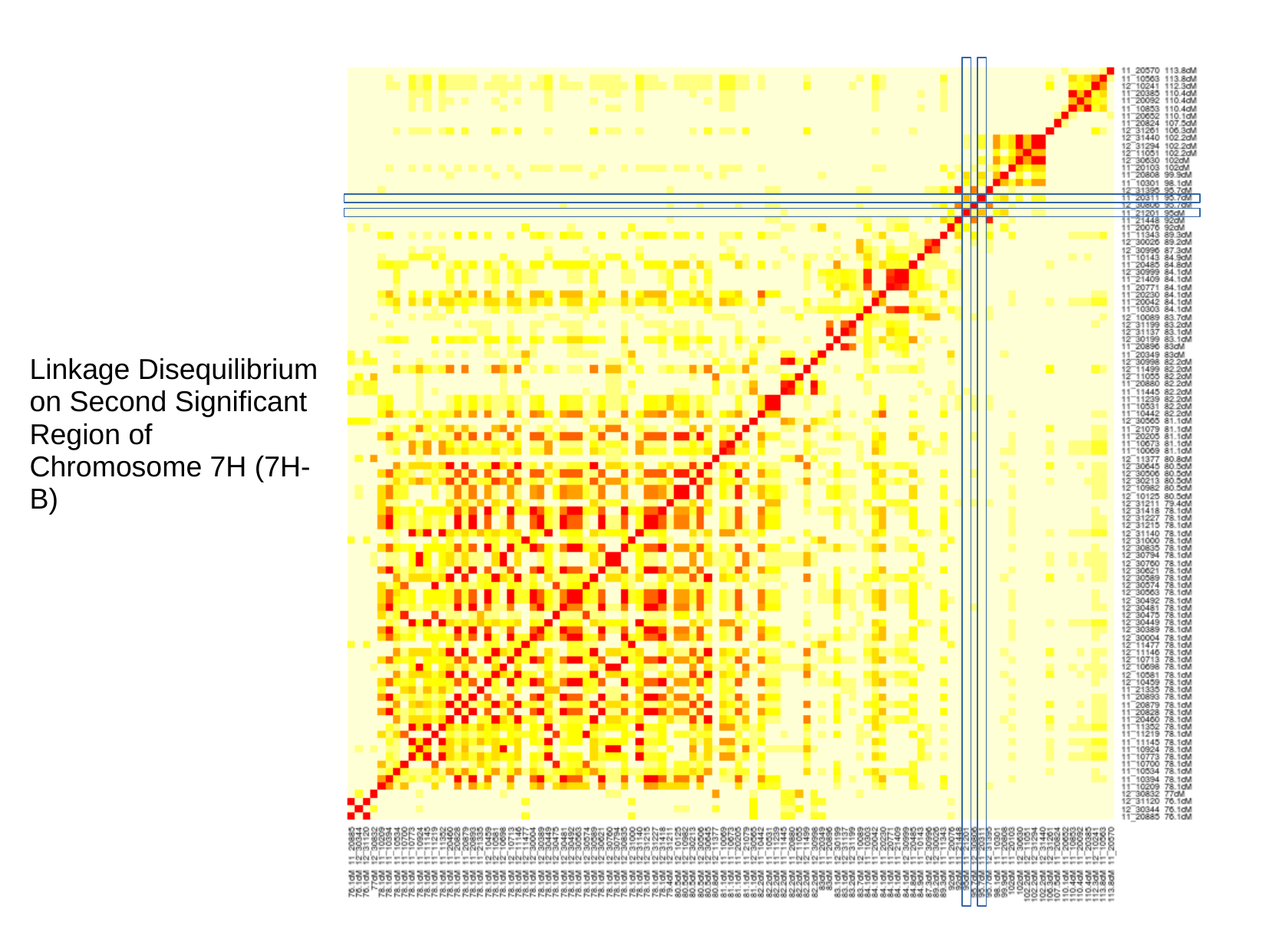

Linkage Disequilibrium on Second Significant Region of Chromosome 7H (7H-B)

## Slide 5
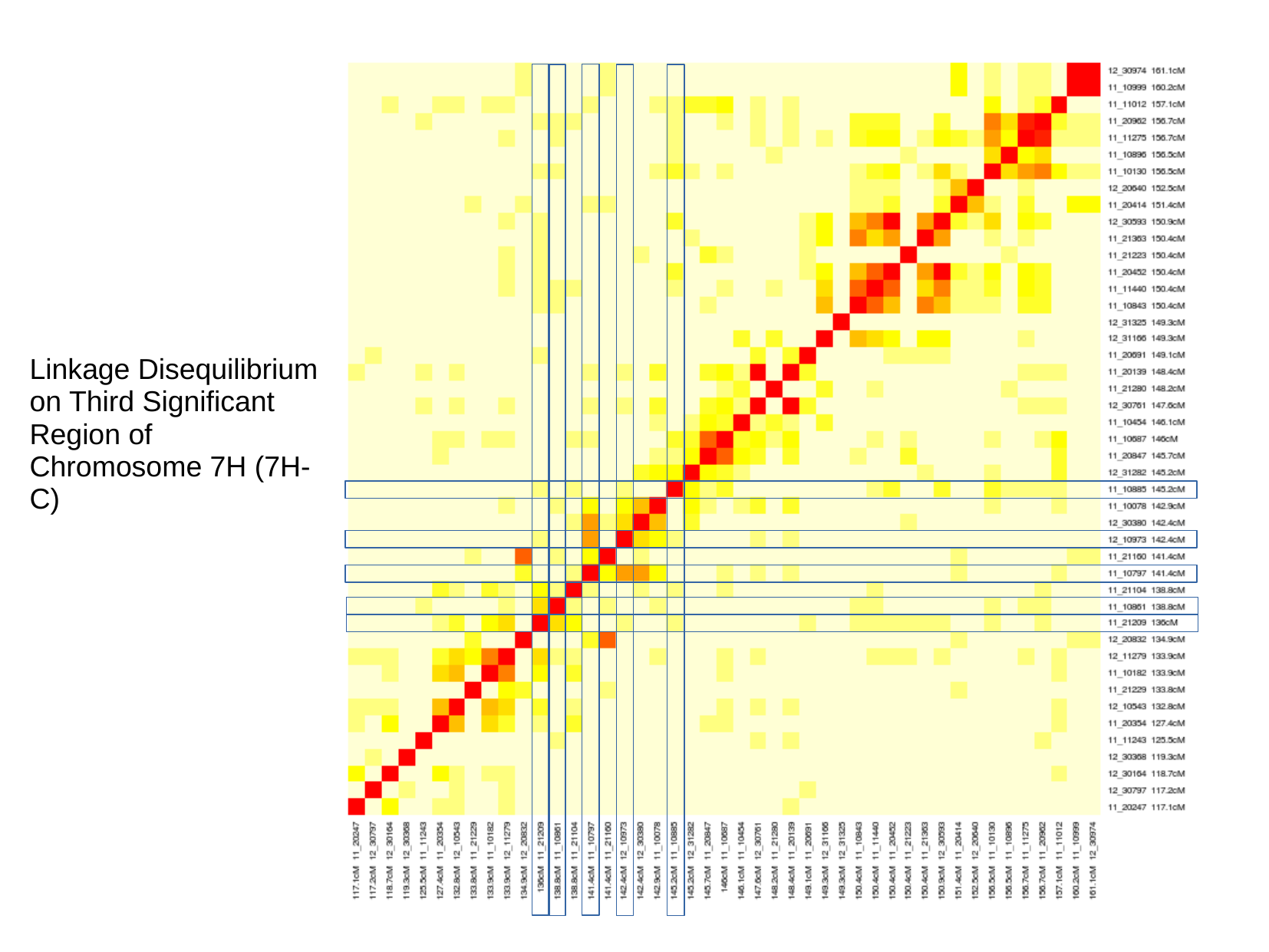

Linkage Disequilibrium on Third Significant Region of Chromosome 7H (7H-C)
