# Supplementary material for: Quantitative Trait Loci Associated with the Tocochromanol (Vitamin E) Pathway in Barley
Source: PLoS One. 2015 Jul 24;10(7):e0133767. doi: 10.1371/journal.pone.0133767 (PMC4514886; doi:10.1371/journal.pone.0133767)
Supplement: S2 File — (PPT) [file pone.0133767.s006.ppt]

## Slide 1
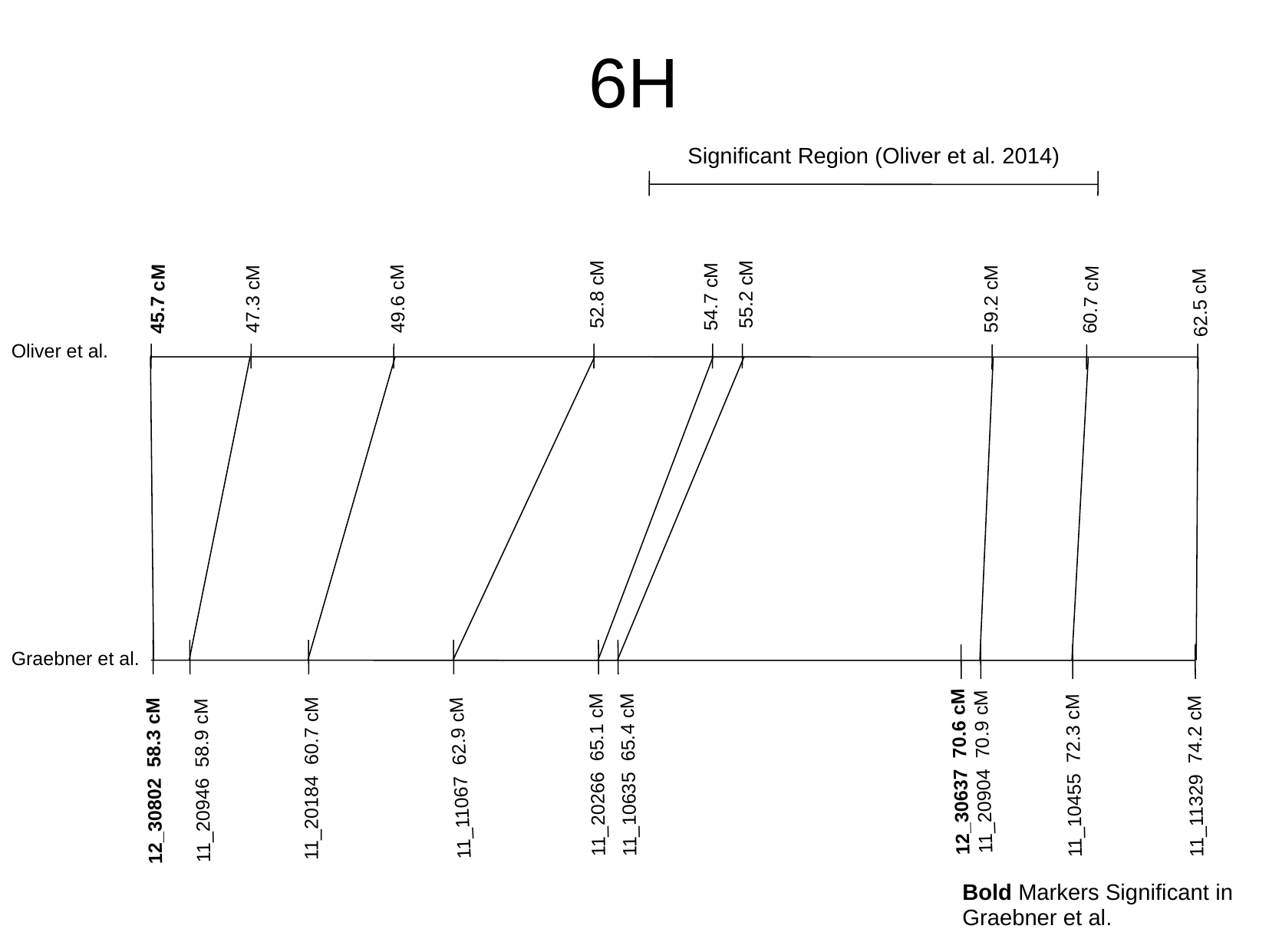

# 6H
Significant Region (Oliver et al. 2014)
52.8 cM
55.2 cM
54.7 cM
45.7 cM
49.6 cM
47.3 cM
59.2 cM
60.7 cM
62.5 cM
Oliver et al.
Graebner et al.
12_30637 70.6 cM
11_20904 70.9 cM
11_20266 65.1 cM
11_10635 65.4 cM
11_10455 72.3 cM
11_11329 74.2 cM
11_11067 62.9 cM
11_20184 60.7 cM
11_20946 58.9 cM
12_30802 58.3 cM
Bold Markers Significant in Graebner et al.

## Slide 2
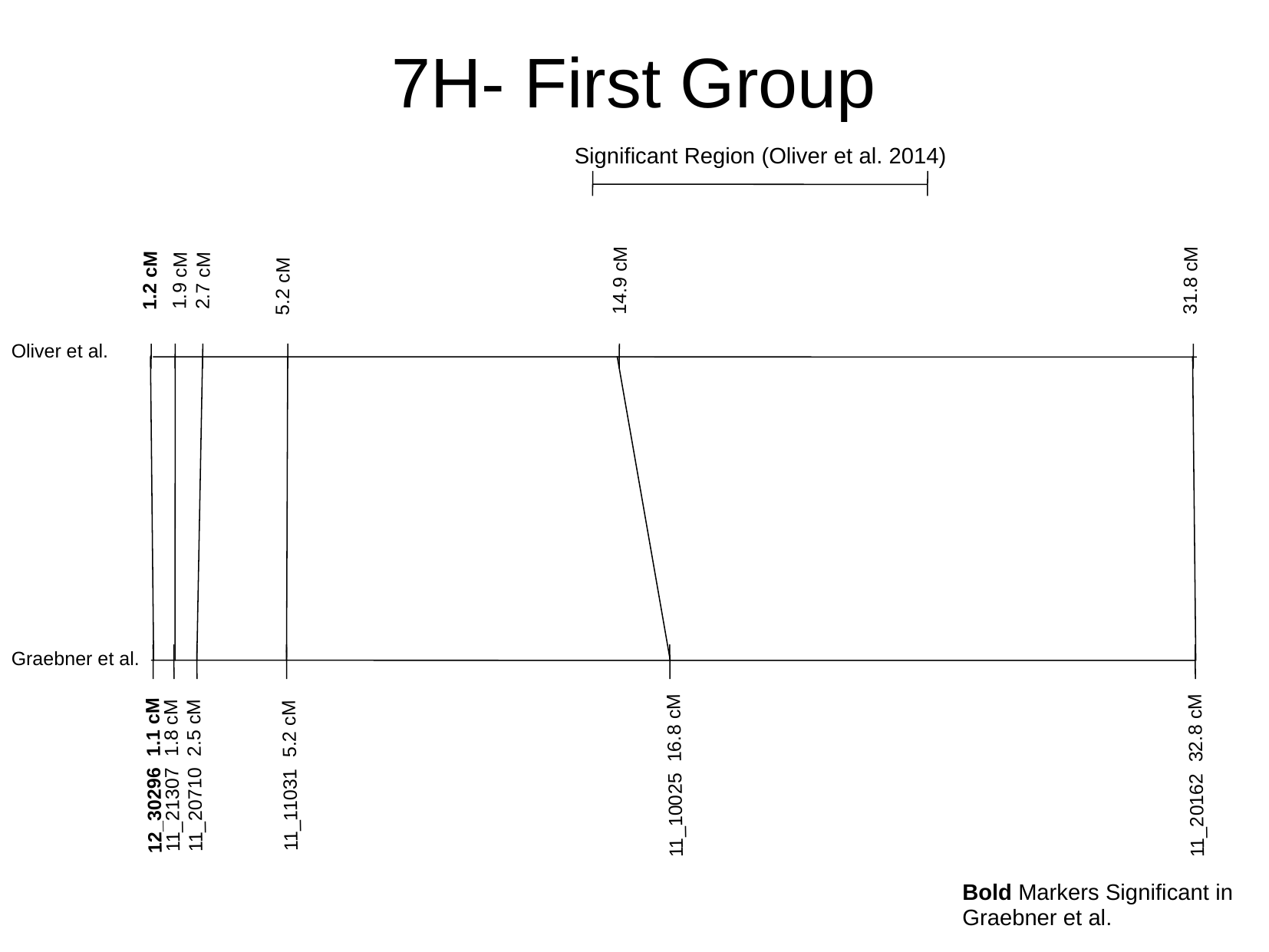

# 7H- First Group
Significant Region (Oliver et al. 2014)
1.2 cM
2.7 cM
1.9 cM
14.9 cM
31.8 cM
5.2 cM
Oliver et al.
Graebner et al.
11_20162 32.8 cM
11_21307 1.8 cM
11_20710 2.5 cM
11_10025 16.8 cM
12_30296 1.1 cM
11_11031 5.2 cM
Bold Markers Significant in Graebner et al.

## Slide 3
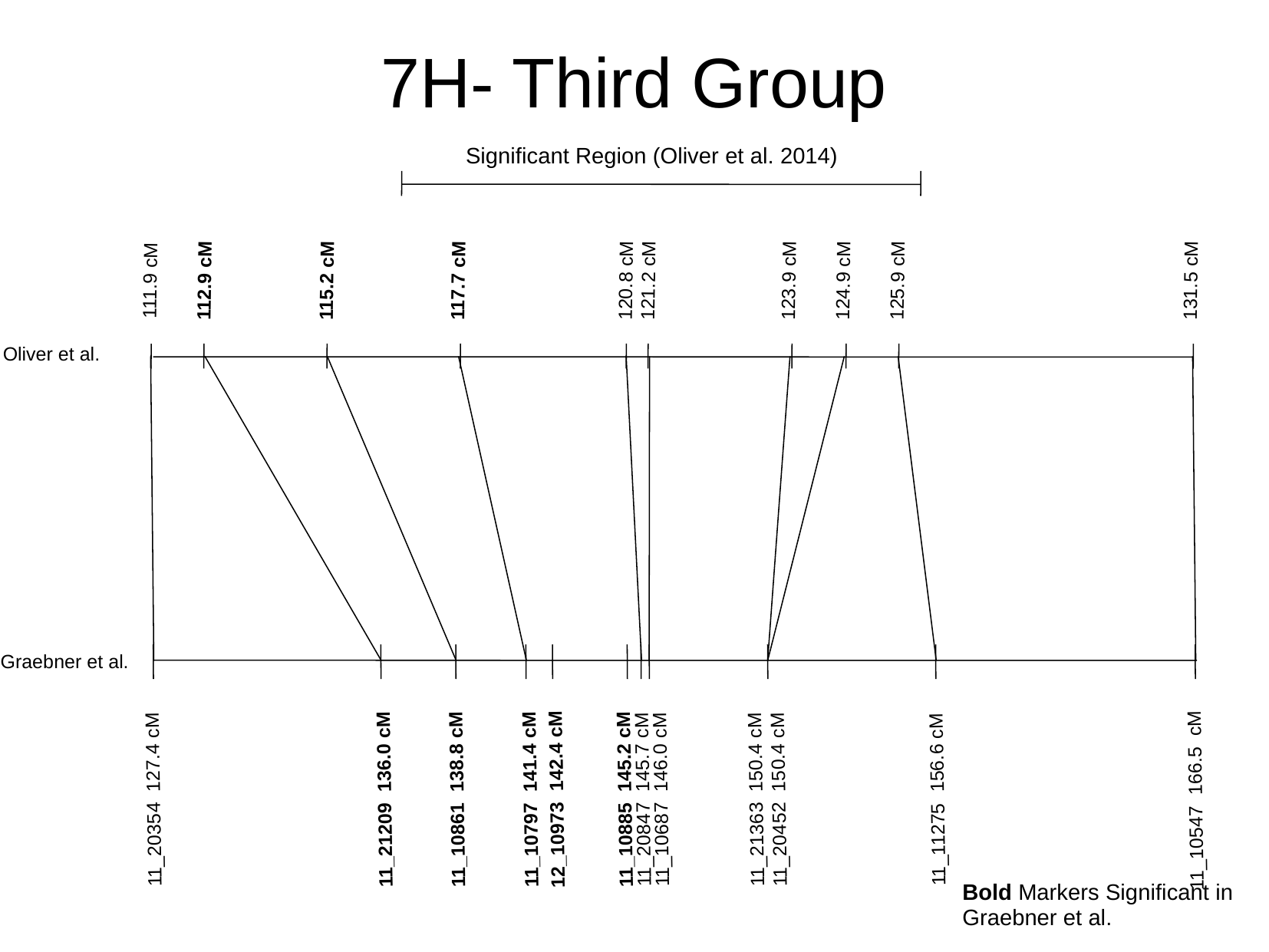

# 7H- Third Group
Significant Region (Oliver et al. 2014)
111.9 cM
112.9 cM
117.7 cM
120.8 cM
121.2 cM
123.9 cM
125.9 cM
131.5 cM
115.2 cM
124.9 cM
Oliver et al.
Graebner et al.
11_20354 127.4 cM
11_21209 136.0 cM
11_10797 141.4 cM
12_10973 142.4 cM
11_21363 150.4 cM
11_20452 150.4 cM
11_10861 138.8 cM
11_10885 145.2 cM
11_20847 145.7 cM
11_10687 146.0 cM
11_11275 156.6 cM
11_10547 166.5 cM
Bold Markers Significant in Graebner et al.
