# Supplementary material for: Quantitative Trait Loci Associated with the Tocochromanol (Vitamin E) Pathway in Barley
Source: PLoS One. 2015 Jul 24;10(7):e0133767. doi: 10.1371/journal.pone.0133767 (PMC4514886; doi:10.1371/journal.pone.0133767)
Supplement: S1 Table — (DOC) [file pone.0133767.s007.doc]

**S1 Table. Analyses of variances for α-tocopherol and TTC concentration in barley.**

| ANOVA Table for αT | |  |  |  |  |
| --- | --- | --- | --- | --- | --- |
| Source | DF | SS | MS | F-value | Pr(>F) |
| Program | 9 | 1438.4 | 159.80 | 42.4 | <0.001 |
| Row Type | 1 | 200.9 | 200.90 | 53.3 | <0.001 |
| Year | 1 | 5533.5 | 5533.50 | 1466.6 | <0.001 |
| Residuals | 1454 | 5485.8 | 3.80 |  |  |
|  |  |  |  |  |  |
| ANOVA Table for αT3 | |  |  |  |  |
| Source | DF | SS | MS | F-value | Pr(>F) |
| Program | 9 | 2527.0 | 280.82 | 11.5 | <0.001 |
| Row Type | 1 | 265.0 | 264.75 | 10.8 | 0.001 |
| Year | 1 | 1772.0 | 1771.94 | 72.6 | <0.001 |
| Residuals | 1454 | 35486.0 | 24.41 |  |  |
|  |  |  |  |  |  |
| ANOVA Table for βT | |  |  |  |  |
| Source | DF | SS | MS | F-value | Pr(>F) |
| Program | 9 | 5.0 | 0.55 | 17.8 | <0.001 |
| Row Type | 1 | 0.9 | 0.91 | 29.3 | <0.001 |
| Year | 1 | 33.3 | 33.32 | 1076.3 | <0.001 |
| Residuals | 1454 | 45.0 | 0.03 |  |  |
|  |  |  |  |  |  |
| ANOVA Table for βT3 | |  |  |  |  |
| Source | DF | SS | MS | F-value | Pr(>F) |
| Program | 7 | 398.2 | 56.89 | 12.2 | <0.001 |
| Row Type | 1 | 4.9 | 4.93 | 1.1 | 0.304 |
| Residuals | 755 | 3521.8 | 4.67 |  |  |
|  |  |  |  |  |  |
| ANOVA Table for δT | |  |  |  |  |
| Source | DF | SS | MS | F-value | Pr(>F) |
| Program | 9 | 3.0 | 0.33 | 20.1 | <0.001 |
| Row Type | 1 | 1.2 | 1.20 | 73.4 | <0.001 |
| Year | 1 | 2.3 | 2.34 | 142.6 | <0.001 |
| Residuals | 1451 | 23.8 | 0.02 |  |  |
|  |  |  |  |  |  |
| ANOVA Table for δT3 | |  |  |  |  |
| Source | DF | SS | MS | F-value | Pr(>F) |
| Program | 9 | 10.4 | 1.16 | 9.4 | <0.001 |
| Row Type | 1 | 0.4 | 0.40 | 3.3 | 0.070 |
| Year | 1 | 129.7 | 129.74 | 1058.0 | <0.001 |
| Residuals | 1452 | 178.1 | 0.12 |  |  |
|  |  |  |  |  |  |
| ANOVA Table for γT | |  |  |  |  |
| Source | DF | SS | MS | F-value | Pr(>F) |
| Program | 7 | 437.7 | 62.52 | 64.9 | <0.001 |
| Row Type | 1 | 224.3 | 224.28 | 232.7 | <0.001 |
| Residuals | 755 | 727.8 | 0.96 |  |  |
|  |  |  |  |  |  |
| ANOVA Table for γT3 | |  |  |  |  |
| Source | DF | SS | MS | F-value | Pr(>F) |
| Program | 9 | 1668.8 | 185.42 | 98.2 | <0.001 |
| Row Type | 1 | 373.7 | 373.68 | 197.9 | <0.001 |
| Year | 1 | 3135.2 | 3135.21 | 1660.4 | <0.001 |
| Residuals | 1454 | 2745.5 | 1.89 |  |  |
|  |  |  |  |  |  |
| ANOVA Table for TTC | |  |  |  |  |
| Source | DF | SS | MS | F-value | Pr(>F) |
| Program | 9 | 21072.0 | 2341.30 | 35.1 | <0.001 |
| Row Type | 1 | 1957.0 | 1956.55 | 29.3 | <0.001 |
| Year | 1 | 1388.0 | 1388.50 | 20.8 | <0.001 |
| Residuals | 1454 | 97001.0 | 66.71 |  |  |
