# Supplementary material for: Quantitative Trait Loci Associated with the Tocochromanol (Vitamin E) Pathway in Barley
Source: PLoS One. 2015 Jul 24;10(7):e0133767. doi: 10.1371/journal.pone.0133767 (PMC4514886; doi:10.1371/journal.pone.0133767)
Supplement: S2 Table — (DOC) [file pone.0133767.s008.doc]

**S2 Table. Distribution of alleles for significant markers associated for tocochromanols for each of eight breeding program.**

| Marker |  | AB | BA | MN | MT | N2 | N6 | UT | WA |
| --- | --- | --- | --- | --- | --- | --- | --- | --- | --- |
|  | Total Individuals | 190 | 186 | 191 | 192 | 183 | 189 | 132 | 190 |
| 11_20021 | A | 104 | 107 | 10 | 192 | 183 | 1 | 32 | 159 |
|  | B | 86 | 79 | 181 | 0 | 0 | 188 | 87 | 31 |
|  | Heterozygous | 0 | 0 | 0 | 0 | 0 | 0 | 4 | 0 |
| 11_10586 | A | 77 | 75 | 1 | 52 | 79 | 21 | 45 | 53 |
|  | B | 112 | 111 | 189 | 140 | 102 | 168 | 81 | 134 |
|  | Heterozygous | 1 | 0 | 1 | 0 | 1 | 0 | 4 | 3 |
| 12_30802 | A | 173 | 149 | 163 | 155 | 182 | 189 | 118 | 90 |
|  | B | 17 | 37 | 28 | 37 | 1 | 0 | 13 | 98 |
|  | Heterozygous | 0 | 0 | 0 | 0 | 0 | 0 | 1 | 2 |
| 12_30637 | A | 105 | 112 | 6 | 189 | 137 | 0 | 91 | 168 |
|  | B | 85 | 74 | 185 | 3 | 40 | 189 | 40 | 22 |
|  | Heterozygous | 0 | 0 | 0 | 0 | 6 | 0 | 1 | 0 |
| 12_30296 | A | 64 | 47 | 0 | 119 | 21 | 1 | 57 | 86 |
|  | B | 122 | 138 | 191 | 71 | 161 | 187 | 75 | 85 |
|  | Heterozygous | 2 | 0 | 0 | 0 | 1 | 1 | 0 | 0 |
| 11_21201 | A | 152 | 167 | 166 | 143 | 161 | 189 | 113 | 186 |
|  | B | 35 | 19 | 24 | 49 | 20 | 0 | 11 | 4 |
|  | Heterozygous | 3 | 0 | 1 | 0 | 2 | 0 | 1 | 0 |
| 11_20311 | A | 32 | 15 | 0 | 26 | 2 | 0 | 11 | 1 |
|  | B | 155 | 171 | 191 | 166 | 180 | 189 | 116 | 189 |
|  | Heterozygous | 3 | 0 | 0 | 0 | 1 | 0 | 3 | 0 |
| 11_21209 | A | 109 | 77 | 175 | 42 | 71 | 145 | 115 | 92 |
|  | B | 79 | 109 | 16 | 150 | 107 | 43 | 15 | 93 |
|  | Heterozygous | 2 | 0 | 0 | 0 | 5 | 0 | 0 | 5 |
| 11_10861 | A | 32 | 98 | 14 | 109 | 74 | 33 | 8 | 68 |
|  | B | 154 | 88 | 177 | 83 | 105 | 155 | 124 | 119 |
|  | Heterozygous | 4 | 0 | 0 | 0 | 4 | 1 | 0 | 3 |
| 11_10797 | A | 169 | 171 | 191 | 143 | 140 | 189 | 68 | 110 |
|  | B | 19 | 15 | 0 | 49 | 39 | 0 | 61 | 74 |
|  | Heterozygous | 2 | 0 | 0 | 0 | 3 | 0 | 2 | 5 |
| 12_10973 | A | 17 | 2 | 0 | 58 | 10 | 0 | 62 | 62 |
|  | B | 172 | 184 | 191 | 134 | 173 | 189 | 70 | 122 |
|  | Heterozygous | 1 | 0 | 0 | 0 | 0 | 0 | 0 | 6 |
| 11_10885 | A | 48 | 78 | 0 | 68 | 61 | 0 | 46 | 46 |
|  | B | 140 | 108 | 191 | 123 | 116 | 189 | 83 | 136 |
|  | Heterozygous | 2 | 0 | 0 | 1 | 6 | 0 | 0 | 6 |
| 12_31511 | A | 2 | 7 | 0 | 36 | 0 | 0 | 31 | 77 |
|  | B | 188 | 179 | 191 | 156 | 183 | 189 | 101 | 109 |
|  | Heterozygous | 0 | 0 | 0 | 0 | 0 | 0 | 0 | 3 |
